# Higher Frequencies of Lymphocytes Expressing the Natural Killer Group 2D Receptor in Patients With Behçet Disease

**DOI:** 10.3389/fimmu.2018.02157

**Published:** 2018-09-25

**Authors:** Martina Bonacini, Alessandra Soriano, Alessandro Zerbini, Eleonora Calò, Luca Cimino, Francesco Muratore, Luigi Fontana, Luca Braglia, Maria Parmeggiani, Carlo Salvarani, Stefania Croci

**Affiliations:** ^1^Unit of Clinical Immunology, Allergy and Advanced Biotechnologies, Azienda Unità Sanitaria Locale-IRCCS di Reggio Emilia, Reggio Emilia, Italy; ^2^Unit of Rheumatology, Azienda Unità Sanitaria Locale-IRCCS di Reggio Emilia, Reggio Emilia, Italy; ^3^Campus Bio-Medico, University of Rome, Rome, Italy; ^4^Unit of Ocular Immunology, Azienda Unità Sanitaria Locale-IRCCS di Reggio Emilia, Reggio Emilia, Italy; ^5^University of Modena and Reggio Emilia, Modena, Italy; ^6^Unit of Ophtalmology, Azienda Unità Sanitaria Locale-IRCCS di Reggio Emilia, Reggio Emilia, Italy; ^7^Research and Statistics Infrastructure, Azienda Unità Sanitaria Locale-IRCCS di Reggio Emilia, Reggio Emilia, Italy

**Keywords:** Behçet disease, NK, NKT, NKG2D, pathogenesis

## Abstract

Behçet disease (BD) is an inflammatory systemic disease with a fluctuating course, which can affect the skin, eyes, central nervous system, musculoskeletal, gastrointestinal, and vascular systems. No laboratory tests are currently available for the diagnosis of BD and monitoring disease activity. Moreover there is a lack of knowledge on BD pathogenesis. This study focused on circulating Natural Killer (NK), NKT and T cells evaluated as CD3^neg^ CD56^pos^, CD3^pos^ CD56^pos^, and CD3^pos^ CD56^neg^. Peripheral blood mononuclear cells (PBMCs) were collected from 38 BD patients and 20 healthy controls (HC). The frequencies of NK, NKT, and T cells expressing CD16, CD69, NKG2D, Nkp30, Nkp46, and NKG2A were assessed by flow cytometry. Cytotoxic potential of NK cells was evaluated by flow cytometry as the percentage of cells expressing the degranulation marker CD107a after incubation with K562 cells. The levels of 27 cytokines were determined in plasma with a multiplex bead-based assay. Higher percentages of NK, NKT, and T cells expressing NKG2D were detected in PBMCs of BD patients than HC. ROC curve analysis showed that the evaluation of NKG2D^pos^ NK, NKT, and T cell percentages discriminated between BD patients and HC. Moreover, there was a positive correlation between the BD Current Activity Form (BDCAF) scores and the frequencies of NKG2D^pos^ NK and NKT cells. A higher frequency of NK cells expressing CD107a was induced in PBMCs from BD patients than HC after incubation with K562 cells. Concentrations of IL-5, IL-6, IL-10, IL-13, IP-10, and MIP-1β were higher in plasma of BD patients than HC. Monitoring the frequencies of NKG2D^pos^ lymphocytes could help the clinicians in BD patients management. In addition, the increased expression of NKG2D in BD patients is likely involved in disease pathogenesis.

## Introduction

Behçet disease (BD) is a rare, systemic, inflammatory chronic disease with multiorgan damage and various clinical manifestations and characterized by alternation of active and remitting phases. BD can affect mucocutaneous, ocular, musculoskeletal, nervous, vascular, gastrointestinal, and cardiac compartments (1). Both genders are affected and the onset is more common in the third decade of life, but in young males the course of disease is more severe (1). Etiopathogenesis is still unknown but genetic and environmental factors are likely implicated in the onset of the disease. Despite HLA-B51 and some gene polymorphisms have been associated with BD (2, 3), the diagnosis is based on a combination of clinical symptoms and signs. Currently no laboratory assays or imaging approaches are available to support the clinical diagnosis. The classification criteria, introduced in 1990 and successively reviewed in 2006, are still a matter of discussion (4), however they help the physicians in identifying the patients with this condition. The BD Current Activity Form (BDCAF) is the most widely used index to assess disease activity in BD (5). BDCAF was developed in 1999 (6) and it scores the presence or absence of clinical features (oral ulcers, genital ulcers, skin lesions, etc.) which were present during the 4 weeks prior to the day of assessment.

Natural killer (NK) cells are cytotoxic lymphocytes able to recognize cells lacking self-MHC class I molecules or cells which display changes in the surface self-molecules. Upon activation, NK cells release cytotoxic granules which contain perforin and granzymes, leading to the destruction of cellular membrane of target cells and subsequently apoptosis (7). Natural killer T (NKT) cells are a lymphocyte subpopulation which express surface molecules characteristic of NK and T cells. Like NK cells, activated NKT cells release in the extracellular compartment pro- and anti-inflammatory cytokines/chemokines with the function of regulating the immune response (8). The activation of NK and NKT cells derives from the balance of signals coming from activatory and inhibitory receptors (9, 10). There are some evidences about the possible involvement of NK and NKT cells in the pathogenesis of BD, but there is a discrepancy in the data. An increased frequency of circulating NK and NKT cells in BD patients compared to healthy controls (HC) has been reported (11–14). Conversely, other authors have reported a decreased frequency of circulating NK and NKT cells in BD patients compared to HC (15, 16).

This study aimed to identify a specific profile of circulating NK, NKT, and T cells able to discriminate between BD patients and HC, investigating the phenotypic characteristics of such cells, the cytotoxic potential of NK cells and quantifying 27 cytokines in plasma. The analysis of peripheral blood NK, NKT and T cells could increase the knowledge about the molecular mechanisms involved in the pathogenesis of BD.

## Materials and methods

### Cohorts of patients and healthy controls

A cohort of 38 BD patients was enrolled at the Azienda Unità Sanitaria Locale-IRCCS, Arcispedale Santa Maria Nuova, Reggio Emilia, Italy. All patients satisfied the International Study Group for Behçet disease criteria (ISGB 1990). For the evaluation of the disease activity, the BDCAF 2006 was administered to the patients during the rheumatologic visit. The median age was 40 years (InterQuartile Range; IQR: 29–50) and gender distribution was: 55% male (21/38) and 45% female (17/38). 74% (28/38) of BD patients were receiving therapies. The characteristics of each BD patient and the type of therapy are summarized in Table S1. 20 age-matched HC were recruited as reference. The median age was 40 (IQR: 33–58) and gender distribution was: 40% (8/20) male and 60% (12/20) female. They did not have any autoimmune diseases, infections, and cancers at the time of blood withdrawal. The median age and gender distribution were similar between the two cohorts. The study was approved by the Local Ethics Committee (Reggio Emilia, Italy, protocol number 2015/0024354) in compliance with the Declaration of Helsinki and written informed consent was obtained from all patients and healthy controls.

### Biological sample collection

18 mL of venous blood were collected from each subject into EDTA coated tubes. Peripheral blood mononuclear cells (PBMCs) were isolated by histopaque-1077 density gradient centrifugation (Sigma-Aldrich) and stored frozen in liquid nitrogen in 90% heat inactivated fetal bovine serum (FBS, Gibco, ThermoFisher) 10% dimethyl sulfoxide (DMSO, Sigma-Aldrich) until use. Plasma was collected and stored at −80°C until use.

### Flow cytometry

PBMCs were thawed and counted with a Fuchs-Rosenthal hemocytometer. The viability of thawed PBMCs was evaluated by Trypan Blue assay. 5 × 10^5^ cells were suspended in 100 μL Phosphate-Buffered Saline (PBS, Euroclone) + 1% FBS and stained for 25 min at 4°C with the following antibodies: PerCP mouse anti-human CD3 (clone BW264/56), PE anti-human CD56 (clone REA196), FITC anti-human CD16 (clone REA423), PE-Vio770^Tm^ mouse anti-human CD69 (clone FN50), and APC mouse anti-human NKG2D (clone BAT221). Alternatively, 5 × 10^5^ cells/100 μL PBS + 1% FBS were stained for 25 min at 4°C with PerCP mouse anti-human CD3 (clone BW264/56), PE anti-human CD56 (clone REA196), PE-Vio770^Tm^ anti-human NKG2A (clone REA110), APC mouse anti-human Nkp30 (clone AF29-4D12), VioBright^Tm^ FITC mouse anti-human Nkp46 (clone 9E2) antibodies. All antibodies were purchased from Miltenyi Biotec and used as suggested by the manufacturer. After washing, PBMCs were suspended in PBS + 1% FBS and acquired with the FACSCanto II flow cytometer (BD Biosciences), equipped with two lasers for excitation at 488 and 633 nm. Data were analyzed with FACSDiva 8.0.1 software. At least 60,000 lymphocytes were acquired. Gates were defined using fluorescence minus one (FMO) controls (Figure S1). To be sure that freezing and defrost process did not differentially altered the percentages of NK, NKT, and T cells and the expression of the surface markers, results obtained with fresh PBMCs were compared with those obtained with thawed PBMCs from BD patients vs. HC. The freezing/defrost process did not modify the percentages of NK, NKT, and T lymphocytes and the percentages of cells positive for the investigated surface markers with the exception of a reduction >10% in the frequencies of CD69^pos^ NK, NKG2A^pos^ T, Nkp46^pos^ NK cells in all the samples. Since such reductions were similar in thawed PBMCs from BD patients and HC (Table S2), analyses were performed on thawed PBMCs.

### Cell lines

K562 cell line, a human erythroleukemic cell line which does not express MHC class I molecules, was provided by Dr. Alessandro Zerbini from the Azienda Ospedaliero-Universitaria of Parma, Italy and maintained in RPMI 1640 (Gibco, ThermoFisher) supplemented with 10% FBS, 100 U/mL penicillin (Euroclone), and 100 μg/mL streptomycin sulfate (Euroclone) at 37°C, 5% CO_2_.

### Degranulation assay

PBMCs were thawed, counted with a Fuchs-Rosenthal hemocytometer and suspended at a density of 2 × 10^6^ cell/mL in RPMI 1640 supplemented with 10% FBS, 100 U/mL penicillin and 100 μg/mL streptomycin sulfate. After overnight incubation at 37°C, 5% CO_2_ with or without 1 ng/mL IL-15 (Miltenyi Biotec), 5 × 10^5^ PBMCs were incubated with K562 target cells, at an effector to target ratio of 5:1, in presence of mouse anti-human CD107a APC-conjugate antibody (clone H4A3, Miltenyi Biotec) for 1 h at 37°C, 5% CO_2_. Then, 10 μg/mL brefeldin A (Sigma-Aldrich) and 6 μg/mL monensin (Sigma-Aldrich) were added to the cells and incubation was carried out for additional 3 h at 37°C, 5% CO_2_. Cells were first stained with 100 μL Live/Dead Fixable Dead Cell Stain near-IR-fluorescent reactive dye (Life Technologies) at 0.1% in PBS for 15 min at room temperature and then with the antibodies against the surface antigens CD3 and CD56 diluted in 100 μL of PBS + 1% FBS for 25 min at 4°C. After washing, cells were suspended in PBS + 1% FBS and acquired with the FACSCanto II flow cytometer. Gates were defined using FMO controls (Figure S2). To be sure that freezing and defrost process did not differentially altered the percentage of CD107a NK cells, results obtained with fresh PBMCs were compared with those obtained with thawed PBMCs from BD patients vs. HC. The freezing/defrost process reduced more than 10% the frequencies of CD107a^pos^ NK cells in thawed PBMCs in all the samples. Since such reductions were similar in thawed PBMCs from BD patients and HC (Table S2), analyses were performed on thawed PBMCs.

### Cytokine assay

Concentrations of IL-1β, IL-1ra, IL-2, IL-4, IL-5, IL-6, IL-7, IL-8, IL-9, IL-10, IL-12p70, IL-13, IL-15, IL-17A, Eotaxin, Basic FGF, G-CSF, GM-CSF, INF-γ, IP-10, MCP-1, MIP-1α, MIP-1β, PDGF-BB, RANTES, TNF-α, and VEGF were determined in plasma of BD patients and HC by the Bio-Plex Pro Human Cytokine Group I Panel, 27-Plex (Biorad) following the manufacturer's instruction. Plasma was diluted four-fold in Bio-Plex Sample Diluent as recommended. Data were obtained with the MAGPIX™ Multiplex Reader instrument and Bio-Plex^®^ Manager^TM^ software. Values extrapolated from the standard curve were considered not reliable and a concentration = 0.01 pg/mL was arbitrary assigned (Table S3 for the lower limits of detection).

### Statistical analysis

Statistical analyses were performed with GraphPad Prism 7 software. For comparisons between two groups non-parametric Mann-Whitney U test was used for quantitative variables, while Fisher's exact test was used for qualitative variables. To adjust for multiple testing, in addition to individual *p*-values, we used the Benjamini and Hochberg method, to control for a false-discovery-rate < 10%. Spearman test was used for correlations between two variables and receiver operating characteristic (ROC) curve was used to assess the performance of a binary classifier system. The performances of the combination of ROC curves were evaluated via logistic regression and Area Under the Curve (AUC) estimation (with 95% confidence interval). AUCs were then compared with z test using R 3.5.0. *P* < 0.05 (two-tailed) were considered statistically significant.

## Results

### Circulating NK, NKT, and T cells percentages in BD patients and HC

**Figure 1:**
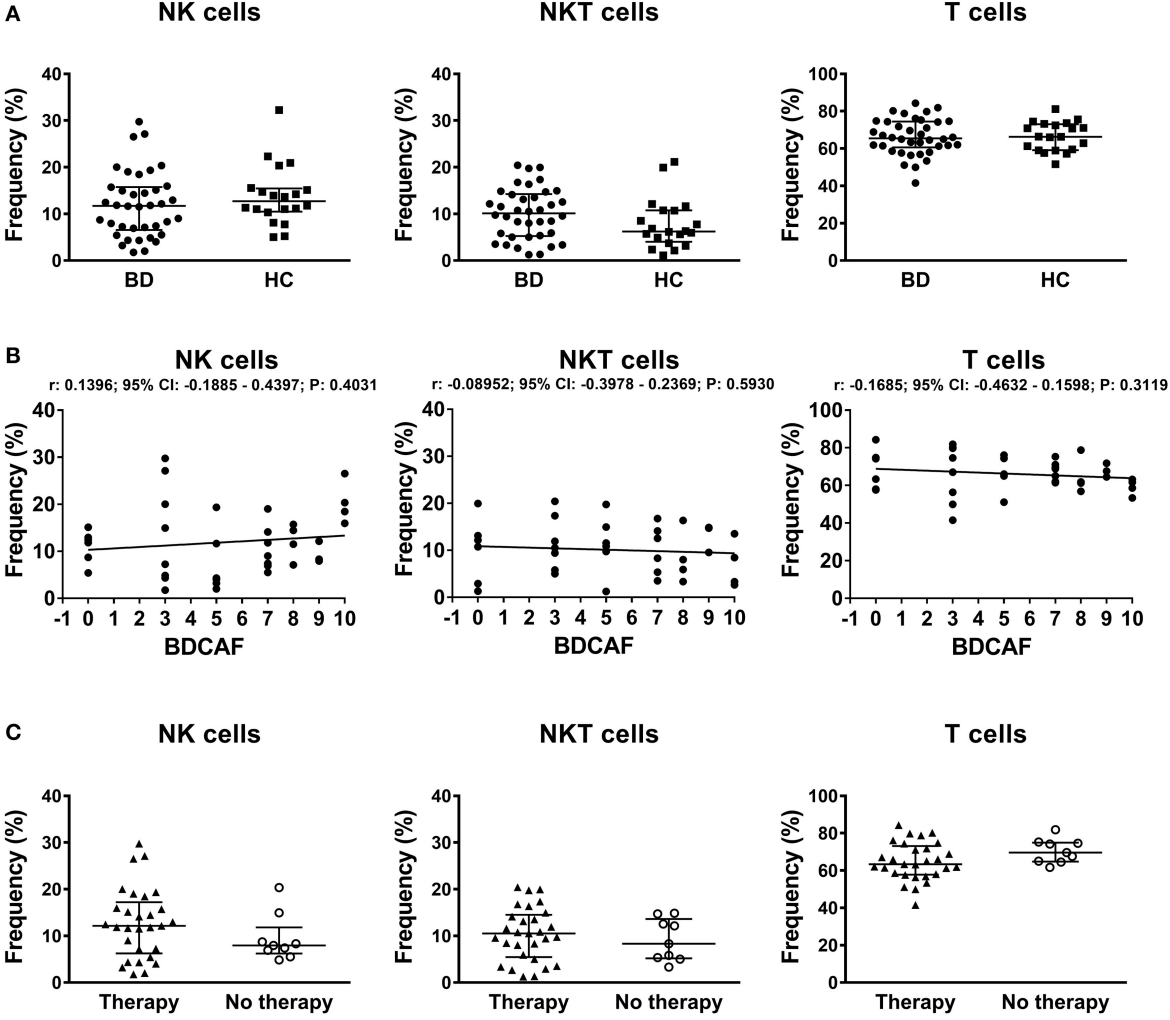
PBMC subsets in BD patients and HC. **(A)** Dot plot visualization of the percentages of NK, NKT, and T cells in the lymphocyte gate by flow cytometry in PBMCs from BD patients (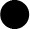) and HC (■). Horizontal lines show the median ± InterQuartile Range (IQR). Data were analyzed by Mann-Whitney U test. **(B)** Dot plot visualization of the correlation between the frequencies of NK, NKT, and T cells and BDCAF scores for each BD patient. Data were analyzed by Spearman's correlation test (*n* = 38). **(C)** Dot plot visualization of the percentages of NK, NKT and T cells determined by flow cytometry in PBMCs from BD patients classified according to presence (▴) or absence (◦) of therapy. Horizontal lines show the median ± IQR. Data were analyzed by Mann-Whitney U test.

Gating strategy for identification of lymphocyte subsets is shown in Figure S1. In the lymphocyte gate NK, NKT, and T cells were defined as CD3^neg^ CD56^pos^, CD3^pos^ CD56^pos^, and CD3^pos^ CD56^neg^ respectively. The median viability of thawed PBMCs were similar between BD patients and HC: 96.1% (IQR: 92.8–97.2%) vs. 96.0% (IQR: 93.9–97.1%). No differences were found in the percentages of NK, NKT, T cells in peripheral blood between BD patients and HC (Figure 1A). The classification of BD patients based on BDCAF scores did not reveal any correlations with the percentages of NK, NKT, and T cells (Figure 1B). Moreover, we did not find any differences in the percentages of NK, NKT, and T cells if BD patients were classified according to the presence (*n* = 28) or absence (*n* = 10) of therapy (Figure 1C). The percentages of CD56^bright^ NK cells were similar between BD patients and HC (data not shown).

### Surface markers of NK and NKT cells in BD patients and HC

**Figure 2:**
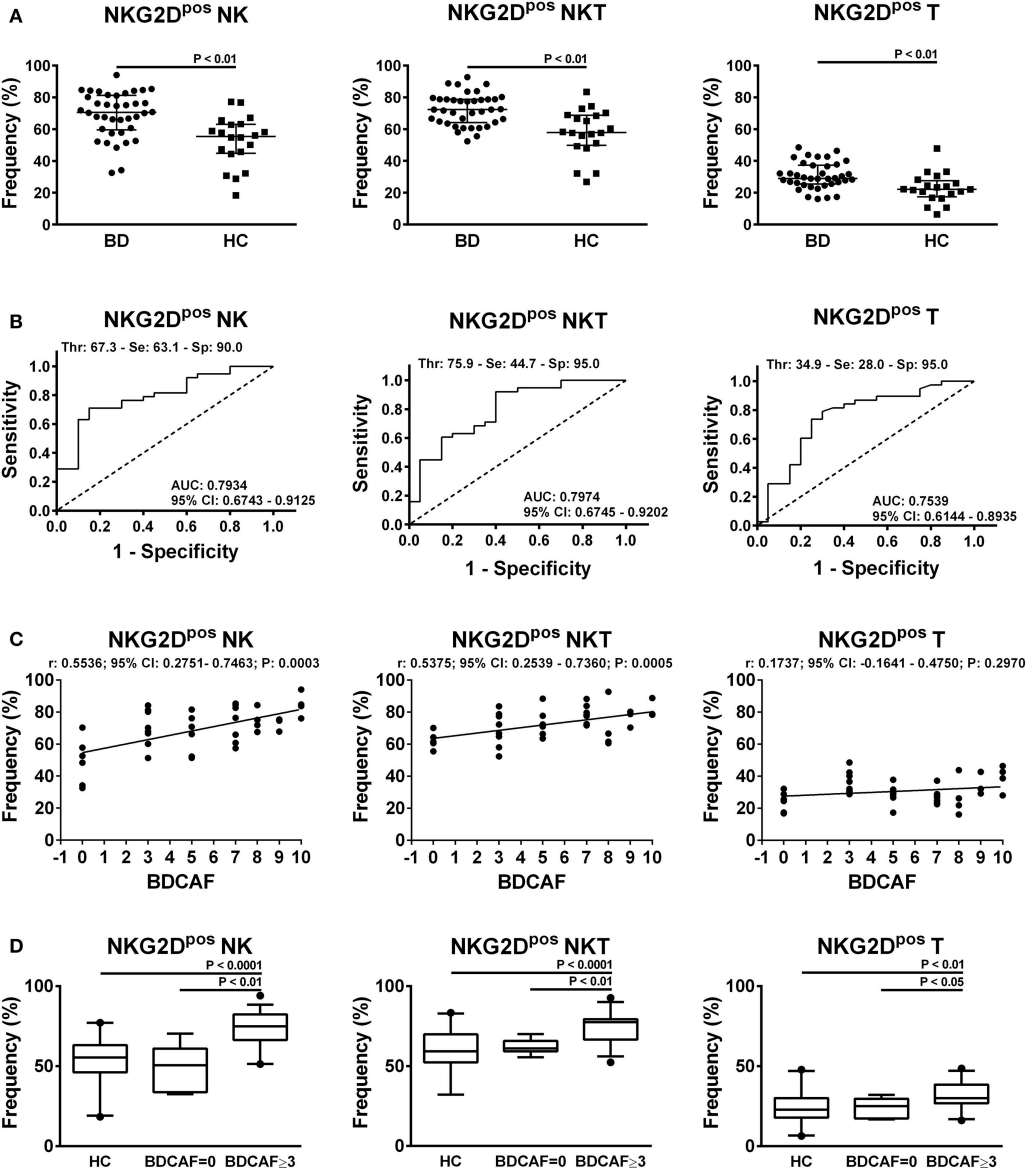
NKG2D expression in lymphocytes from BD patients and HC. **(A)** Dot plot visualization of the percentages of NKG2D^pos^ cells in the NK, NKT, and T lymphocyte gate determined by flow cytometry in PBMCs from BD patients (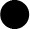) and HC (■). Horizontal lines show the median ± IQR. Data were analyzed by Mann-Whitney U test. **(B)** ROC curve analysis of the percentages of NKG2D^pos^ cells in NK, NKT, and T lymphocyte gates. AUC, Area Under the Curve; CI, Confidence Interval; Thr, Threshold; Se, Sensitivity; Sp, Specificity. **(C)** Dot plot visualization of the correlation between the frequencies of NKG2D^pos^ NK, NKT, and T cells and BDCAF scores for each BD patient. Data were analyzed by Spearman's correlation test (*n* = 38). **(D)** Box plot visualization of the frequencies of NKG2D^pos^ NK, NKT, and T cells in BD patients with BDCAF = 0 vs. BDCAF ≥ 3 and HC. Data were analyzed by Mann-Whitney U test.

In order to characterize the immunophenotype of circulating lymphocytes in BD patients, the frequencies of cells expressing the activatory markers CD69, CD16, NKG2D, Nkp30, Nkp46 and the inhibitory marker NKG2A were analyzed within each lymphocyte subset compared to HC (refer to Figure S1 for the gating strategy). We observed a significant increase in the frequencies of NKG2D^pos^ NK, NKT, and T cells in BD patients with respect to HC (Figure 2A). In particular, the median frequency of NKG2D^pos^ NK cells was 70.6% (IQR: 59.5–81.2%) in BD vs. 55.4% (IQR: 44.8–63.1%) in HC; while the median frequency of NKG2D^pos^ NKT cells was 72.3% (IQR: 64.1–78.8%) in BD vs. 58% (IQR: 49.8–68.8%) in HC and the median frequency of NKG2D^pos^ T cells was 29.5% (IQR: 25.4–37.4%) in BD vs. 22.2% (IQR: 17.4–27.7%) in HC. After adjustment for multiple testing, the increase in the frequencies of NKG2D^pos^ NK, NKT, and T cells in BD patients remained statistically significant.

ROC curve analysis showed that the evaluation of the percentage of NKG2D^pos^ cells in the lymphocyte gate allowed to discriminate between BD patients and HC with low-medium sensitivity but high specificity (Figure 2B for details). No significant differences in AUCs were observed when the ROC curve based on the percentage of NKG2D^pos^ NK cells was compared with the ROC curves combining the percentages of NKG2D^pos^ NK+T cells (*P* = 0.627) or NKG2D^pos^ NK+T+NKT cells (*P* = 0.547) (Figure S3).

**Figure 3:**
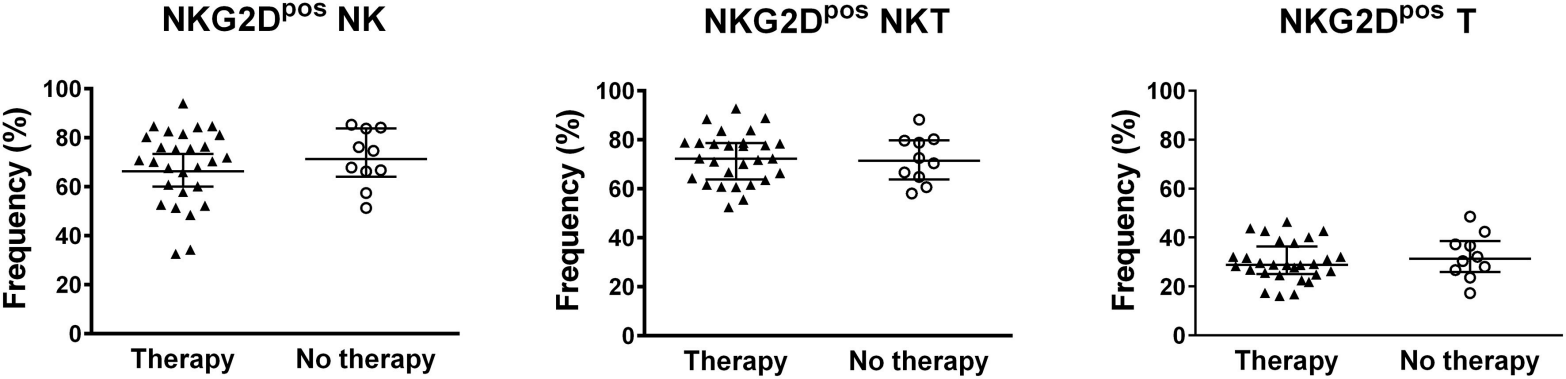
NKG2D expression in lymphocytes of BD patients classified according to the therapy. Dot plot visualization of the percentages of NKG2D^pos^ cells in the NK, NKT, and T lymphocyte gates determined by flow cytometry in PBMCs from BD patients classified according to presence (▴) or absence (◦) of therapy. Horizontal lines show the median ± IQR. Data were analyzed by Mann-Whitney U test.

The classification of BD patients based on BDCAF scores revealed a direct correlation with the frequencies of NKG2D^pos^ NK and NKT cells, while no correlation was observed with the frequency of NKG2D^pos^ T cells (Figure 2C). The subsequent division of patients with BDCAF = 0 (*n* = 6) and BDCAF ≥ 3 (*n* = 32) showed a significant higher frequencies of NKG2D^pos^ NK, NKT, and T cells in patients with BDCAF ≥ 3 in comparison with patients with BDCAF = 0 and HC (Figure 2D). The frequencies of NKG2D^pos^ NK, NKT, and T cells of BD patients with BDCAF = 0 were similar to the frequencies detected in HC (Figure 2D). Instead, the classification of patients according to the presence/absence of therapy did not show any differences in the percentages of NKG2D^pos^ NK, NKT and T cells (Figure 3).

**Figure 4:**
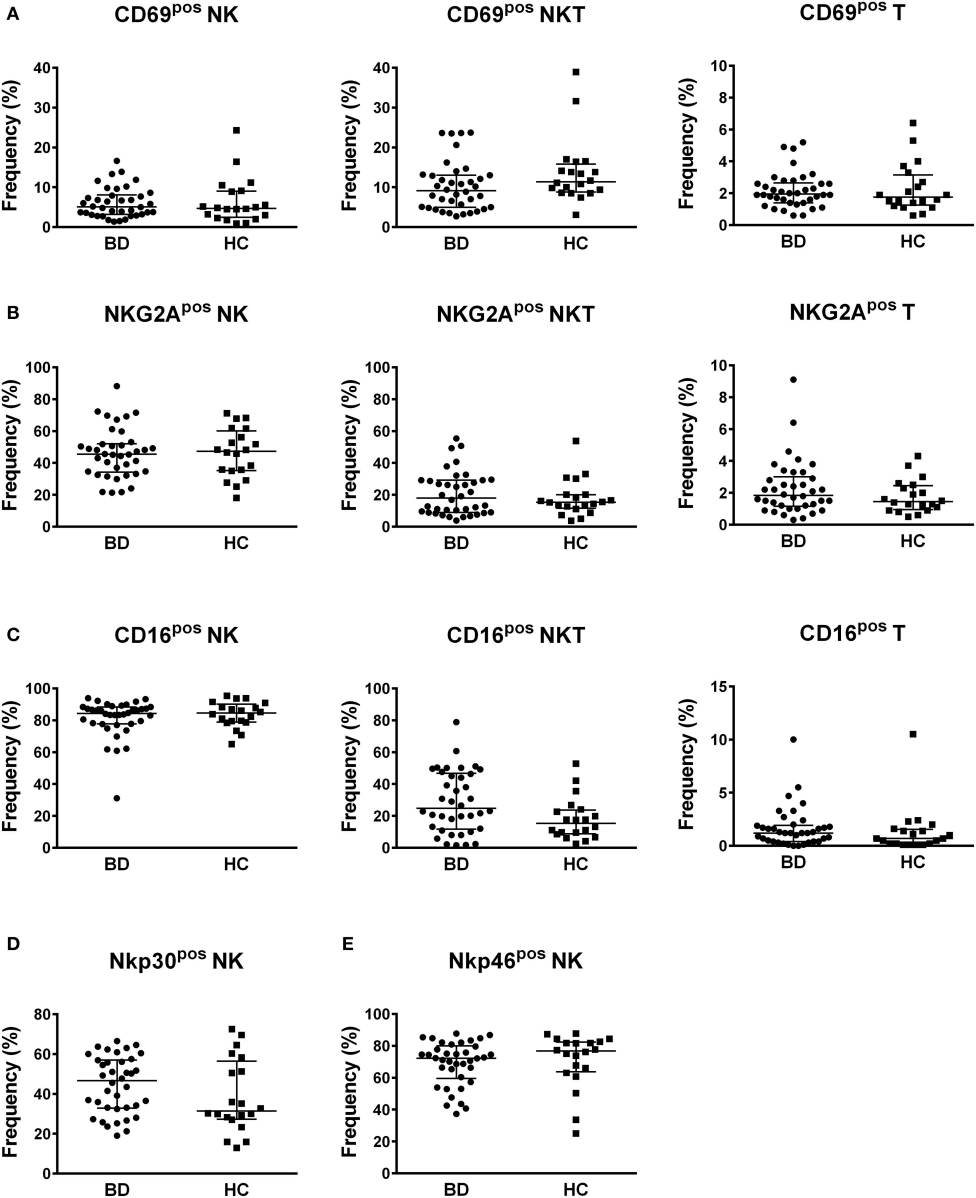
Profile of markers of activation and inhibition. Dot plot visualization of the percentages of CD69 **(A)**, NKG2A **(B)**, CD16 **(C)**, Nkp30 **(D)**, and Nkp46 **(E)** positive cells in NK, NKT and T lymphocyte gates determined by flow cytometry in PBMCs from BD patients (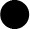) and HC (■). Horizontal lines show the median ± IQR. Data were analyzed by Mann-Whitney U test.

Concerning the frequencies of NK, NKT, and T cells positive for the other inhibitory and activatory surface markers, no differences were found between BD patients and HC (Figure 4). Neither the classification of BD patients based on BDCAF scores nor the classification according to the presence/absence of therapy showed any differences in the percentages of NK, NKT, and T cells expressing the CD69, CD16, Nkp30, Nkp46, and NKG2A surface markers (Figure S4, S5).

### Cytotoxic potential of NK cells

**Figure 5:**
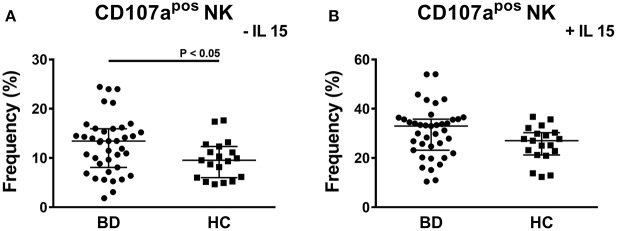
CD107a degranulation assay of PBMCs from BD patients and HC. Dot plot visualization of the percentages of CD107a^pos^ cells in the NK lymphocyte gate determined by flow cytometry in PMBCs from BD patients (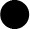) and HC (■) after stimulation with K562 in presence of 10 μg/mL brefeldin A and 6 μg/mL monensin. The assay was conducted without **(A)** or with 1ng/mL IL-15 **(B)** to maximize the stimulus. Horizontal lines show the median ± IQR. Data were analyzed by Mann-Whitney U test.

**Figure 6:**
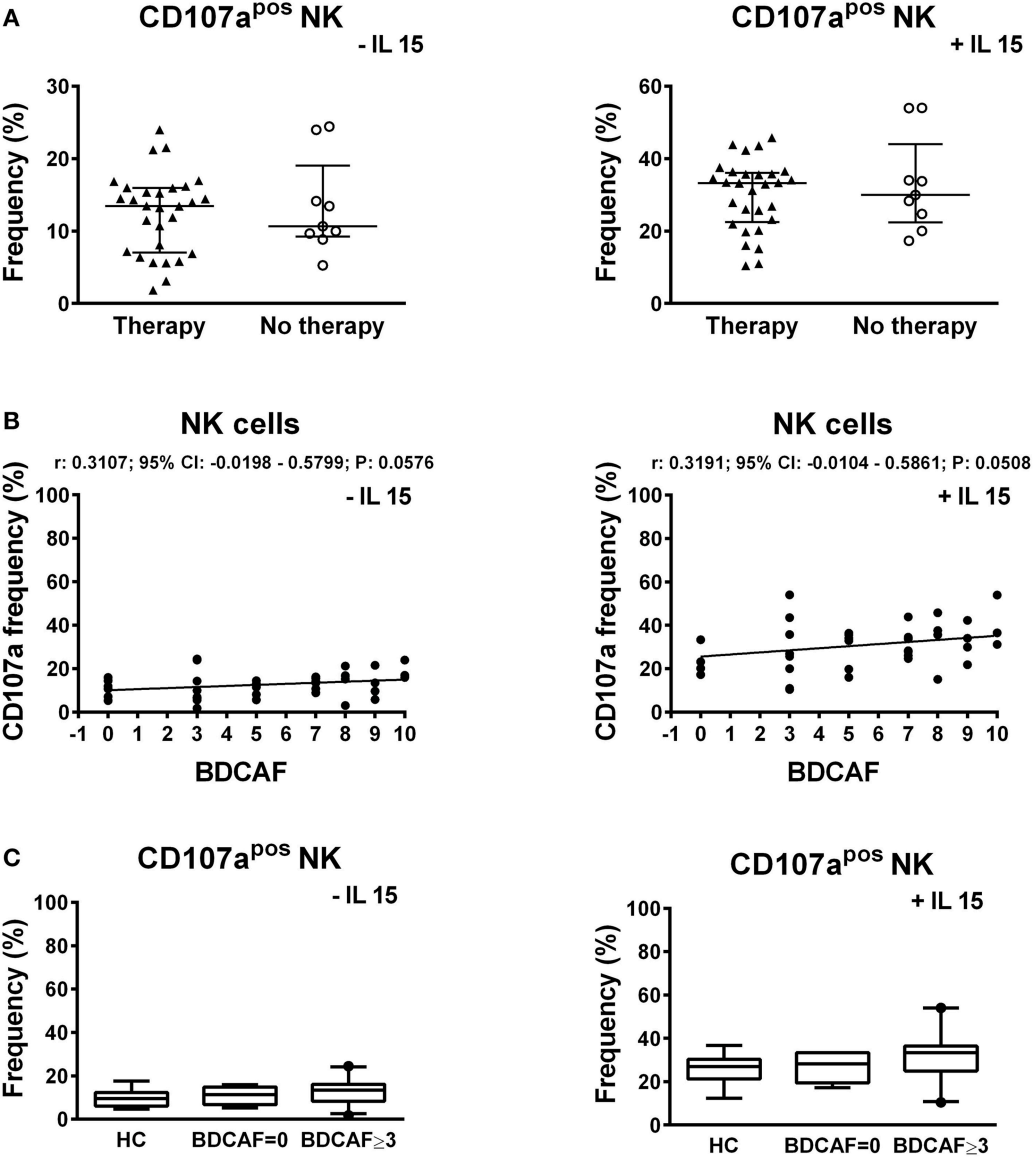
CD107a expression in lymphocytes of BD patients classified according to the BDCAF scores and therapy. **(A)** Dot plot visualization of the percentages of CD107a^pos^ NK cells determined by flow cytometry in PBMCs from BD patients classified according to presence (▴) or absence (◦) of therapy, after stimulation with K562 in presence of 10 μg/mL brefeldin A and 6 μg/mL monensin. Horizontal lines show the median ± IQR. Data were analyzed by Mann-Whitney U test. **(B)** Dot plot visualization of the correlation between the frequencies of CD107a^pos^ NK cells and BDCAF scores for each BD patient. Data were analyzed by Spearman's correlation test (*n* = 38). **(C)** Box plot visualization of the frequencies of CD107a^pos^ NK cells in BD patients with BDCAF = 0 vs. BDCAF ≥ 3 and HC. Data were analyzed by Mann-Whitney U test.

To study the cytotoxic potential of NK cells, PBMCs of BD patients and HC were stimulated through contact with K562 cells, which do not express MHC-I, followed by the analysis of the expression of the CD107a degranulation marker. The stimulus with K562 cells is specific for NK cells and it does not activate the cytotoxic potential of NKT and T cells. A significant higher frequency of CD107a^pos^ NK cells was induced in BD patients compared to HC: 13.43% (IQR: 8.09–15.95%) vs. 9.54% (IQR: 6–12.33%), *P* = 0.0314 (Figure 5A). In particular, a bimodal distribution appeared in the cohort of BD patients with 5 patients showing a higher frequency of CD107a^pos^ NK cells. We were not able to find any commonalities in such subjects (e.g., presence/absence of therapy, type of therapy, organ involvement, disease activity, sex, age, HLA-B51 status). Further stimulation of PBMCs with IL-15 increased the percentages of CD107a^pos^ cells, but no differences were found between BD patients and HC (Figure 5B). Neither the classification of BD patients based on the BDCAF scores nor the classification according to the presence/absence of therapy revealed any differences in the cytotoxic potential of NK cells (Figure 6).

### Cytokine plasmatic levels in BD patients and HC

Multiplex analysis of 27 cytokines using plasma samples revealed a significantly higher concentration of IL-4, IL-5, IL-6, IL-10, IL-13, IL-17A, IP-10, MCP-1, and MIP1β in BD patients in comparison to HC (Table 1). After adjustment for multiple testing, the increase of IL-5, IL-6, IL-10, IL-13, IP-10, and MIP1β in plasma of BD patients remained statistically significant. No differences were found in BD patients classified on BDCAF scores (Table S4), while the classification according to presence/absence of therapy revealed a higher concentration of IP-10 in plasma of BD patients without therapy in comparison to patients under therapy: 835.5 pg/mL (IQR: 570.7–1398 pg/mL) vs. 446.3 pg/mL (IQR: 297.6–676.1 pg/mL), *P* = 0.0023. The concentration of IP-10 in plasma of BD patients with therapy was similar to the concentration detected in HC (319 pg/mL; IQR: 310.7–484.1). Instead, the concentration of IP-10 in plasma of BD patients without therapy was significantly higher than that of HC (*P* < 0.0001). Some cytokines were detected only in a fraction of subjects and the differences of frequencies of detection were statistically significant between BD patients and HC in the case of IL-6, IL-10, and IL-13 (Table 1). In particular, IL-6 was detected in 15/38 (39%) of BD patients and 2/20 (10%) of HC, IL-10 was detected in 19/38 (50%) of BD patients, and 4/20 (20%) of HC, while IL-13 was detected in 38/38 (100%) of BD patients and 15/20 (75%) of HC.

**Table 1:** Levels of cytokines in plasma of BD patients and HC.

	**Concentration (pg/mL)**			**Positive subjects (fraction)**		
	**BD *n* = 38**	**HC *n* = 20**	**Mann-Whitney U test *P* value**	**BD *n* = 38**	**HC *n* = 20**	**Fisher's test *P* value**
IL-1β	0.01 (0.01–4.73)	0.01 (0.01–0.01)	0.0701	16/38	4/20	0.1460
IL-1ra	53.83 (23.53–188.40)	32.29 (16.86–80.24)	0.0830	32/38	17/20	1
IL-2	0.01 (0.01–0.01)	n.d.	0.2875	4/38	0/20	0.2875
IL-4	1.78 (0.01–3.55)	0.01 (0.01–0.01)	0.0385^*^	24/38	8/20	0.1055
IL-5	13.13 (4.55–29.49)	5.16 (0.01–17.04)	0.0193^*^	30/38	11/20	0.0734
IL-6	0.01 (0.01–13.43)	0.01 (0.01–0.01)	0.0180^*^	15/38	2/20	0.0318^*^
IL-7	3.20 (0.01–12.51)	0.01 (0.01–3.56)	0.0505	20/38	6/20	0.1643
IL-8	5.94 (0.01–15.68)	0.01 (0.01–7.19)	0.1895	25/38	11/20	0.5700
IL-9	13.66 (3.11–47.59)	8.75 (4.84–16.42)	0.3514	32/38	18/20	0.7015
IL-10	3.58 (0.01–26.73)	0.01 (0.01–0.01)	0.0107^*^	19/38	4/20	0.0467^*^
IL-12 (p70)	7.50 (0.01–27.64)	0.01 (0.01–13.63)	0.0624	20/38	6/20	0.1643
IL-13	8.13 (4.16–12.54)	2.79 (0.38–6.24)	0.0007^***^	38/38	15/20	0.0034^**^
IL-15	0.01 (0.01–0.01)	n.d.	0.3103	3/38	0/20	0.5443
IL-17A	8.74 (0.01–48.59)	0.01 (0.01–10.60)	0.0420^*^	23/38	7/20	0.0973
Eotaxin	67.73 (49.59–105.80)	65.53 (51.11–81.06)	0.4979	38/38	20/20	1
Basic FGF	32.02 (8.50–70.16)	28.40 (12.27–43.76)	0.5661	31/38	17/20	1
G-CSF	19.24 (9.22–61.32)	21.42 (9.51–27.81)	0.6876	32/38	18/20	0.7015
GM-CSF	31.63 (8.12–59.66)	70.10 (0.01–123.80)	0.2402	31/38	14/20	0.3387
IFN-γ	44.57 (0.01–171.6)	21.73 (0.01–54.92)	0.1801	26/38	13/20	0.7784
IP-10	573.40 (356.80–799.60)	319.00 (310.70–484.10)	0.0134^*^	38/38	20/20	1
MCP-1	0.01 (0.01–37.76)	0.01 (0.01–0.01)	0.0267^*^	18/38	4/20	0.0506
MIP-1α	3.62 (2.20–6.17)	2.70 (1.87–4.14)	0.1475	37/38	19/20	1
MIP-1β	40.62 (23.35–88.57)	27.25 (20.01–34.36)	0.0213^*^	38/38	20/20	1
PDGF-BB	104.90 (28.62–289.3)	75.69 (27.00–135.80)	0.4096	37/38	20/20	1
RANTES	1972 (1156–4954)	1467 (980.30–3138)	0.1930	38/38	20/20	1
TNF-α	21.57 (0.01–92.34)	12.88 (0.01–21.68)	0.0620	25/38	11/20	0.5700
VEGF	12.58 (0.01–32.93)	6.34 (0.01–17.41)	0.0810	28/38	11/20	0.2386

Cytokine concentrations are expressed in pg/ml as median (IQR). Data were analyzed by Mann-Whitney U test. Fractions represent the number of subjects in which the cytokines were detectable. A cytokine was considered not detectable if its signal was lower than that of the least concentrated cytokine standard. Data were analyzed by Fisher's exact test. n.d., not detected;

* P < 0.05;

** P < 0.01;

*** *P < 0.001*.

## Discussion

The present work contributes to expand the knowledge about the mechanisms underlying Behçet disease. For the first time, we reported an increased frequencies of circulating NK, NKT, and T cells positive for the activatory surface marker NKG2D in BD patients compared to HC. Differently from other authors, we did not find any differences in the frequencies of circulating NK, NKT, and T lymphocytes in BD patients compared to HC (12–16). The clinical heterogeneity of the patients (e.g., regarding pre-existing therapy, disease activity and pattern of organ involvement), different ethnicity and different surface markers used for lymphocyte identification could explain the discrepancy of the results obtained so far in BD patients.

NKG2D is a homodimeric C-type lectin-like activating receptor that is expressed on almost all NK and CD8^pos^ T cells and with less frequency on the surface of NKT and CD4^pos^ T cells. NKG2D acts as a sensor for recognition of induced-self antigens in cells infected by pathogens, transformed or stressed cells (17). In humans, 8 different ligands able to bind the NKG2D receptor with different affinity are known: MHC class I chain-related protein A (MICA) and protein B (MICB) and 6 HCMV UL16 binding proteins. Consequent the binding of ligands, NKG2D receptor can initiate an intracellular signal cascade that leads to NK and NKT activation (18). Conversely in T cells the function of NKG2D receptor is different acting as a co-stimulatory molecule enhancing T-cell receptor activation (TCR) and T cells functions (19). In human beings some polymorphisms in MICA gene have been documented. In particular, in patients with BD the polymorphisms in MICA gene: ^*^009 and TM A6 have been found more frequently and are in linkage disequilibrium with HLA-B51 (20, 21). There is not a definitive consensus on whether polymorphisms in MICA gene or HLA-B51 or both have a causal role in the onset of BD. The consequences of MICA polymorphism at protein level (e.g., protein turnover, conformation, interaction with ligands) are still under debate and need to be uncovered to understand their possible involvement in disease pathogenesis.

NKG2D expression has been discovered as altered in different autoimmune diseases. In rheumatoid arthritis an increased percentage of NKG2D^pos^ CD4^pos^ T cells has been reported in blood and synovial fluid (22), while patients affected by systemic lupus erythematosus (SLE) showed an increase in the percentage of NKG2D^pos^ T cells and a decrease of NKG2D in NK cells in terms of expression, percentage, and relative number (23–25). Concerning Behçet disease, two studies identified a reduction in the frequencies of NKG2D^pos^ cells in γδ T lymphocytes and CD8^bright^ CD56^pos^ and CD8^bright^ CD56^neg^ lymphocytes (26, 27), whereas an increased percentage of NKG2D^pos^ CD4^pos^ lymphocytes has been reported by Clemente et al. (28). In our knowledge this is the first study that describes an increased frequencies of circulating NKG2D^pos^ NK, NKT, and T cells. We classified the lymphocytes widely, based on CD3 and CD56 expression thus comparing and integrating the results is difficult. Here, we found that determining the frequencies of circulating NKG2D^pos^ NK, NKT, and T cells could discriminate between BD patients and HC with high degree of specificity, but low sensitivity. The comparison with other inflammatory diseases which need a differential diagnosis with BD is necessary to confirm the diagnostic utility of testing the expression of NKG2D. The BDCAF scores of BD patients correlated with the frequencies of NKG2D^pos^ NK and NKT cells in peripheral blood. Interestingly, BD patients with BDCAF = 0 had a NKG2D profile similar to HC, while BD patients with BDCAF ≥ 3 had a higher frequency of NKG2D^pos^ NK, NKT, and T cells. If NKG2D expression has a role in BD pathogenesis and disease activity we might thus speculate that only patients with BDCAF = 0 might be considered inactive/in remission. In line to our findings, the percentages of NKG2D^pos^ lymphocytes have been previously associated with disease activity in CD8^bright^ CD56^pos^, CD8^bright^ CD56^neg^, and in CD4^pos^ lymphocytes (27, 28).

The comparison between patients with BD and healthy subjects can especially provide knowledge on the pathogenesis of the disease. As a result to the increased frequencies of circulating NKG2D^pos^ NK and NKT cells, the immune system of BD patients might be more prone to respond to ligands of NKG2D when exposed to tissue cells (e.g., in case of cell stress, infections, cell transformation) likely leading to recurrent episodes of inflammation.

Recently, Hasan and collaborators reported an increase of the cytotoxicity of NK cells in peripheral blood of BD patients (15). They utilized the stimulus with phorbol-12-myristate-13-acetate (PMA) and ionomycin to activate NK cells and the cytotoxic potential of the cells was evaluated by monitoring the frequency of NK cells positive for CD107a surface marker. Here, we similarly evaluated the frequency of CD107a^pos^ NK cells but following stimulation by the contact with cells missing the MHC-I class molecules (K562 cells). In comparison to PMA and ionomycin, it is a stimulus that better reflects the physiologic activation of NK cells in the organism. Also in these experimental conditions, the frequency of CD107a^pos^ NK cells was higher in BD patients than HC. This difference mainly derived from 5 patients showing a higher frequency of CD107a^pos^ NK cells. We cannot compare our results with those from Hasan and collaborators because they depicted the degranulation assay results as column graphs which do not allow to evaluate data distribution.

All the cytokines, we found increased in plasma of BD patients, are linked with NK and NKT activity based on literature data. IL-5, IL-10, and IL-13 can be released from activated NK and NKT cells (29, 30). IL-6 can play a role in the activation of NK cells (31). MIP-1β, also known as CCL4, has a double role: to one hand it acts as a NK cell chemoattractant signal produced by dendritic cells; to the other hand it is produced by activated NK cells (32). IP-10, also known as CXCL10, is produced by dendritic cells and can attract NK cells in the sites of inflammation (32).

Increased levels of circulating MIP-1β are reported here for the first time. Differently from our findings, Aktas Cetin et al. did not find any differences regarding the concentration of IL-5 between BD patients and HC (33). According to our findings, increased levels of CXCL10/IP-10 have been reported by Takeuchi et al. (34) while no differences have been found by Lopalco et al. (35) and Saruhan-Direskeneli et al. (36). Literature data regarding IL-10 levels in plasma and/or serum are indeed controversial. In one study lower serum and plasma levels of IL-10 have been reported in BD patients compared with HC (37). No differences have been found in 4 studies (33, 38–40). IL-10 was not detected in serum samples in two studies (41, 42). In line with our results, higher levels of IL-10 have been reported in BD patients by Turan et al. (43), Aridogan et al. (44) and Hamzaoui et al. (45). Concerning IL-6, the involvement of this cytokine pathway in BD is well documented and recently an antibody anti-IL6 receptor blocking IL-6 activities has shown efficacy in the treatment of BD (46). Interestingly, increased levels of circulating IL-13 in BD patients have been reported in other 3 manuscripts suggesting that IL-13 might have a role in BD (44, 47, 48).

Finally, concerning IL-13 and MIP-1β, it is important to highlight that the transcription of these factors is directly under the control of NKG2D pathway (49). Therefore high levels of IL-13 and MIP-1β found in plasma of BD patients might be downstream the increased activation of NKG2D pathway in NK, NKT and T cells of BD patients compared to HC.

The limits of the present study concern the number of patients and the fact that 74% of patients were under heterogeneous therapies. However it has to be considered that Behçet is a rare disease and our study is monocentric. In spite this limits, the strong points of the study is that our cohort represents real life patients thus the increased frequency of NKG2D^pos^ NK, NKT, and T cells probably represents a pathogenic hit of BD.

Taken together, data here reported support the hypothesis that through an increased frequency of NKG2D activatory receptor on the cell surface, NK, NKT and T cells of BD patients could be more prone to respond to NKG2D ligands when exposed on tissue cells, leading to cyclic auto-inflammation. Concerning the clinical practice, monitoring the frequencies of lymphocytes expressing NKG2D could help the clinicians to identify BD patients and/or to confirm disease activity during the follow-up.

## Ethics statement

The study was approved by the Local Ethics Committee (Reggio Emilia, Italy, protocol number 2015/0024354) in compliance with the Declaration of Helsinki and written informed consent was obtained from all patients and healthy controls.

## Author contributions

All authors were involved in drafting the article or revising it critically for important intellectual content, and all authors approved the final version to be published. MB and AS contributed equally to the study. CS and SC shared senior authorship. MB and SC wrote the manuscript. MB, AS, SC, and AZ designed the experimental protocol. AS, LC, FM, and LF recruited patients. MB, SC, AZ, and EC performed the experiments. MB, AS, SC, AZ, MP, LB, and CS interpreted the data.

### Conflict of interest statement

The authors declare that the research was conducted in the absence of any commercial or financial relationships that could be construed as a potential conflict of interest.
