# Supplementary material for: Higher Frequencies of Lymphocytes Expressing the Natural Killer Group 2D Receptor in Patients With Behçet Disease
Source: Front Immunol. 2018 Sep 25;9:2157. doi: 10.3389/fimmu.2018.02157 (PMC6167483; doi:10.3389/fimmu.2018.02157)
Supplement: Supplementary file 1 [file Table_1.pdf]

Table S1. Characteristics of the cohort of BD patients

	District involvement							Disease activity	
	**Mucocutaneous**	**Ocular**	**Gastrointestinal**	**Musculoskeletal**	**Nervous system**	**Vascular**	**HLA-B51 status**	**BDCAF score**	**Therapy**
**Pz#1**	X	X					Positive	10	None
**Pz#2**	X	X					Positive	10	Steroid
**Pz#3**	X	X					Positive	8	Steroid + DNA synthesis inhibitor
**Pz#4**	X		X	X	X		Negative	8	Steroid
**Pz#5**	X					X	n.d.	7	Cell cycle inhibitor
**Pz#6**		X					Positive	3	Steroid + TNFα inhibitor
**Pz#7**		X					Positive	8	DNA synthesis inhibitor
**Pz#8**	X	X					Positive	7	Steroid
**Pz#9**	X	X				X	Negative	7	None
**Pz#10**	X	X					Positive	3	Transcription inhibitor
**Pz#11**		X					Positive	5	IFNα
**Pz#12**	X	X		X			n.d	10	Steroid
**Pz#13**	X	X		X			Positive	9	None
**Pz#14**	X					X	n.d	7	Cell cycle inhibitor
**Pz#15**		X					Positive	3	None
**Pz#16**	X	X		X			Negative	5	Steroid
**Pz#17**	X	X					Positive	7	None
**Pz#18**	X			X			Positive	9	None
**Pz#19**	X			X		X	n.d.	9	Steroid + Cell cycle inhibitor + Transcription inhibitor
**Pz#20**	X	X					n.d	0	Cell cycle inhibitor
**Pz#21**	X					X	n.d.	0	Transcription inhibitor
**Pz#22**	X		X	X		X	Positive	3	DNA synthesis inhibitor + TNFα inhibitor

	District involvement							Disease activity	
	**Mucocutaneous**	**Ocular**	**Gastrointestinal**	**Musculoskeletal**	**Nervous system**	**Vascular**	**HLA-B51 status**	**BDCAF score**	**Therapy**
**Pz#23**	X						n.d.	5	Cell cycle inhibitor
**Pz#24**	X	X			X	X	n.d.	3	DNA synthesis inhibitor
**Pz#25**	X		X	X			Negative	10	Steroid + DNA synthesis inhibitor
**Pz#26**	X			X			n.d.	7	None
**Pz#27**	X	X		X	X	X	n.d.	3	Steroid + Cell cycle inhibitor
**Pz#28**	X	X			X		n.d.	0	TNFα inhibitor
**Pz#29**	X		X	X		X	n.d.	8	None
**Pz#30**		X					n.d.	0	None
**Pz#31**	X	X		X		X	Positive	5	DNA synthesis inhibitor
**Pz#32**	X	X		X		X	n.d.	3	Steroid + TNFα inhibitor
**Pz#33**	X				X		n.d.	0	Steroid + DNA synthesis inhibitor
**Pz#34**	X	X		X		X	n.d.	7	DNA synthesis inhibitor + TNFα inhibitor
**Pz#35**	X			X			n.d.	3	None
**Pz#36**	X			X		X	Positive	0	DNA synthesis inhibitor + Cell cycle inhibitor
**Pz#37**	X				X	X	Positive	5	Steroid + Cell cycle inhibitor
**Pz#38**	X	X		X			Positive	5	Steroid + DNA synthesis inhibitor + TNFα inhibitor

DNA synthesis inhibitor = Mycophenolate mofetil, Azathioprine, Methotrexate;
Cell cycle inhibitor = Colchicine, Cyclophosphamide;
Transcription inhibitor = Ciclosporin;
TNFα inhibitor = Adalimumab, Infliximab.
n.d.= not determined
The district involvement is referred to the historical clinical features of the disease for each patients, while the CAF score is referred to the disease activity at the moment of blood sample collection.

Table S2. Effects of the freezing/defrost process. The frequencies of the cell subsets in thawed PBMCs over fresh PBMCs multiplied per 100 are shown. Data represents the mean ± standard error (SE) from 3 BD patients and 3 HC.

	**BD** Thawed/fresh PBMCs (%)	**HC** Thawed/fresh PBMCs (%)
**NK cells**	98.34 ± 8.25	96.62 ± 1.74
**NKT cells**	92.7 ± 1.18	93.92 ± 3.43
**T cells**	103.5 ± 2.27	102.8 ± 0.71
**NKG2D^{pos} NK cells**	101.2 ± 4.45	98.11 ± 1.30
**NKG2D^{pos} NKT cells**	98.47 ± 0.14	94.06 ± 0.25
**NKG2D^{pos} T cells**	95.99 ± 1.25	93.51 ± 2.91
**CD69^{pos} NK cells**	81.94 ± 7.06	70.27 ± 10.23
**CD69^{pos} NKT cells**	98.51 ± 9.06	96.53 ± 1.80
**CD69^{pos} T cells**	102.5 ± 2.34	88.78 ± 5.53
**NKG2A^{pos} NK cells**	99.01 ± 2.24	99.08 ± 1.89
**NKG2A^{pos} NKT cells**	100.1 ± 4.12	95.66 ± 5.21
**NKG2A^{pos} T cells**	81.44 ± 5.82	78.31 ± 4.13
**CD16^{pos} NK cells**	94.62 ± 2.20	94.09 ± 1.85
**CD16^{pos} NKT cells**	88.6 ± 6.23	92.42 ± 3.79
**CD16^{pos} T cells**	88.25 ± 9.09	90.05 ± 2.52
**Nkp30^{pos} NK cells**	94.03 ± 1.26	91.55 ± 0.95
**Nkp46^{pos} NK cells**	83.73 ± 6.00	81.57 ± 2.84
**CD107a^{pos} NK cells –IL15**	69.77 ± 1.87	75.11 ± 10.82
**CD107a^{pos} NK cells +IL15**	74.24 ± 0.73	71.48 ± 1.82

Table S3. Detection limit of the investigated cytokines. Concentrations lower than these values were considered not reliable and a value = 0.01 was arbitrary assigned.

	Concentration (pg/mL)
**IL-1β**	2.00
**IL-1ra**	16.64
**IL-2**	.68
**IL-4**	1.04
**IL-5**	4.72
**IL-6**	9.20
**IL-7**	3.64
**IL-8**	5.36
**IL-9**	2.44
**IL-10**	6.68
**IL-12 (p70)**	7.48
**IL-13**	1.52
**IL-15**	22.80
**IL-17A**	4.40
**Eotaxin**	6.44
**Basic FGF**	8.12
**G-CSF**	6.28
**GM-CSF**	1.80
**IFN-γ**	6.28
**IP-10**	6.96
**MCP-1**	4.96
**MIP-1α**	0.84
**MIP-1β**	3.02
**PDGF-BB**	1.92
**RANTES**	11.44
**TNF-α**	12.88
**VEGF**	6.52

Table S4. Correlations between cytokine concentrations and BDCAF scores. Data were analysed by Spearman's correlation test (n=38).

	r Spearman	95 % CI	P value
**IL-1β**	-0.2369	-0.5245 to 0.09926	0.1521
**IL-1ra**	-0.2156	-0.5081 to 0.1214	0.1936
**IL-2**	0.04661	-0.2862 to 0.3694	0.7811
**IL-4**	-0.2014	-0.497 to 0.136	0.2253
**IL-5**	-0.07568	-0.3943 to 0.2592	0.6516
**IL-6**	-0.2102	-0.5038 to 0.127	0.2053
**IL-7**	-0.1389	-0.447 to 0.1986	0.4054
**IL-8**	-0.3066	-0.577 to 0.02429	0.0612
**IL-9**	-0.3025	-0.574 to 0.02877	0.0649
**IL-10**	0.07015	-0.2644 to 0.3896	0.6756
**IL-12 (p70)**	0.02333	-0.3075 to 0.3491	0.8894
**IL-13**	0.009536	-0.3199 to 0.3369	0.9547
**IL-15**	-0.2465	-0.5319 to 0.08917	0.1357
**IL-17A**	-0.1904	-0.4883 to 0.1472	0.2521
**Eotaxin**	-0.3937	-0.6395 to -0.07499	0.0568
**Basic FGF**	-0.1692	-0.4715 to 0.1686	0.3098
**G-CSF**	-0.2497	-0.5343 to 0.08577	0.1305
**GM-CSF**	-0.1473	-0.4538 to 0.1904	0.3776
**IFN-γ**	-0.2367	-0.5244 to 0.09949	0.1525
**IP-10**	-0.05066	-0.3729 to 0.2825	0.7626
**MCP-1**	-0.2148	-0.5074 to 0.1223	0.1954
**MIP-1α**	-0.1942	-0.4913 to 0.1434	0.2426
**MIP-1β**	-0.04639	-0.3692 to 0.2864	0.7821
**PDGF-BB**	-0.221	-0.5122 to 0.1159	0.1825
**RANTES**	-0.08923	-0.4058 to 0.2464	0.5942
**TNF-α**	-0.3066	-0.5769 to 0.02433	0.0612
**VEGF**	-0.1051	-0.419 to 0.2314	0.5302
