# Supplementary material for: Higher Frequencies of Lymphocytes Expressing the Natural Killer Group 2D Receptor in Patients With Behçet Disease
Source: Front Immunol. 2018 Sep 25;9:2157. doi: 10.3389/fimmu.2018.02157 (PMC6167483; doi:10.3389/fimmu.2018.02157)

**Figure S1.** Gating strategy. A first gate was set on side-scatter area (SSC-A) *versus* forward-scatter area (FSC-A) to identify lymphocytes. CD3 and CD56 were used to identify NK (CD3$^{neg}$ CD56$^{pos}$), NKT (CD3$^{pos}$ CD56$^{pos}$) and T (CD3$^{pos}$ CD56$^{neg}$) cells within the lymphocyte gate. NK cells = orange dots; NKT cells = red dots; T cells= green dots; CD3$^{neg}$ CD56$^{neg}$ cells = light blue. In the lower panels, it is shown the strategy for the identification of positive/negative cells for NKG2D, NKG2A, CD69, CD16, Nkp30 and Nkp46 markers in NK, NKT and T cell gates. Plots represent overlays of NK, NKT and T sub-gating. Fluorescence minus one (FMO) controls were used to determine positive/negative boundaries.

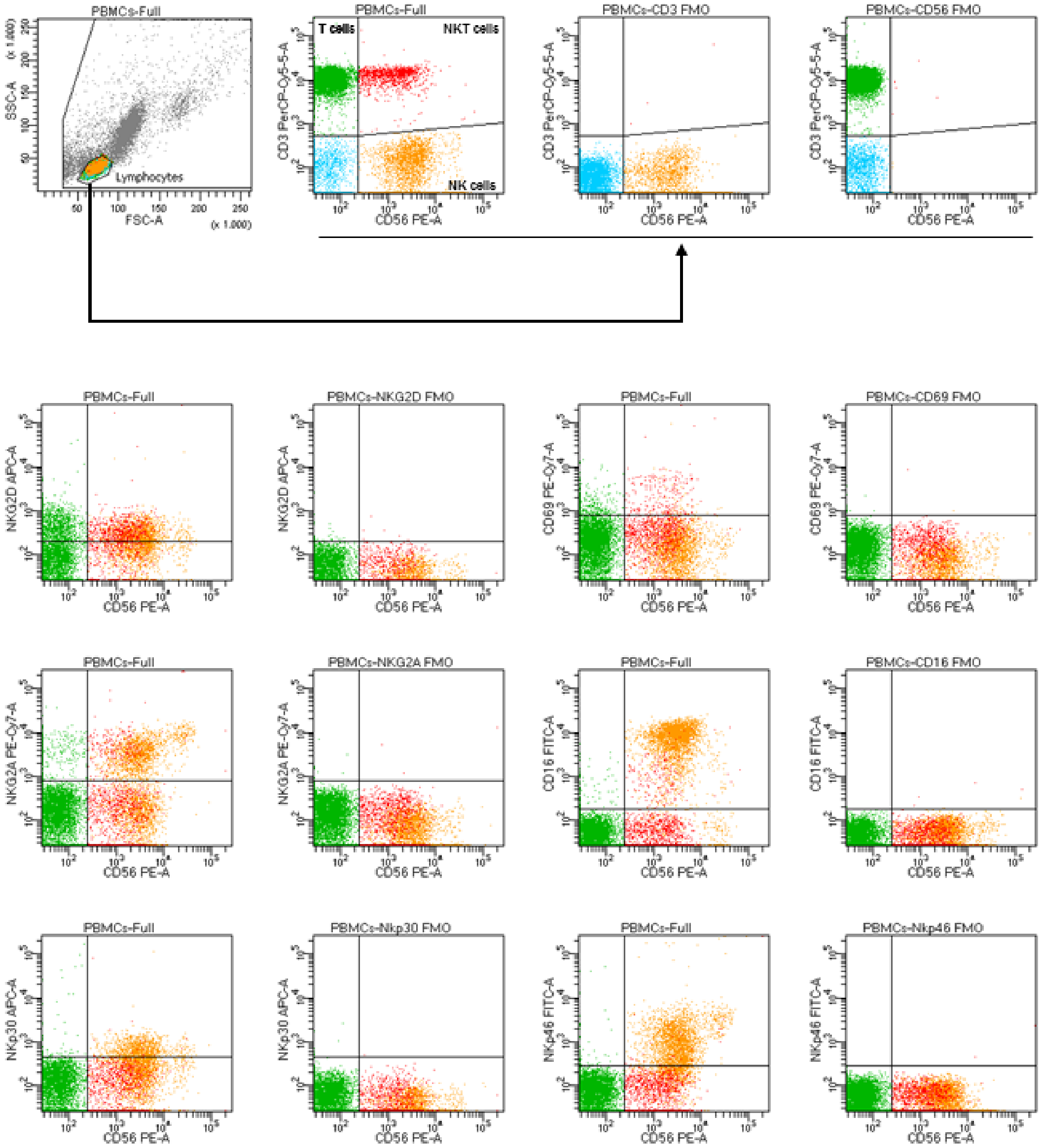

**Figure S2.** Gating strategy for the degranulation assay. A first gate was set on side-scatter area (SSC-A) *versus* forward-scatter area (FSC-A) to identify lymphocytes. A second gate based on Live/Dead staining was set to determine live lymphocytes. CD3 and CD56 were used to classify NK ($CD3^{neg}$ $CD56^{pos}$) cells within the lymphocyte gate (NK = orange dots). CD107a positive/negative NK cells were identified in the NK lymphocyte gate based on CD56-CD107a plot. Fluorescence minus one (FMO) control was used to determine positive/negative boundaries.

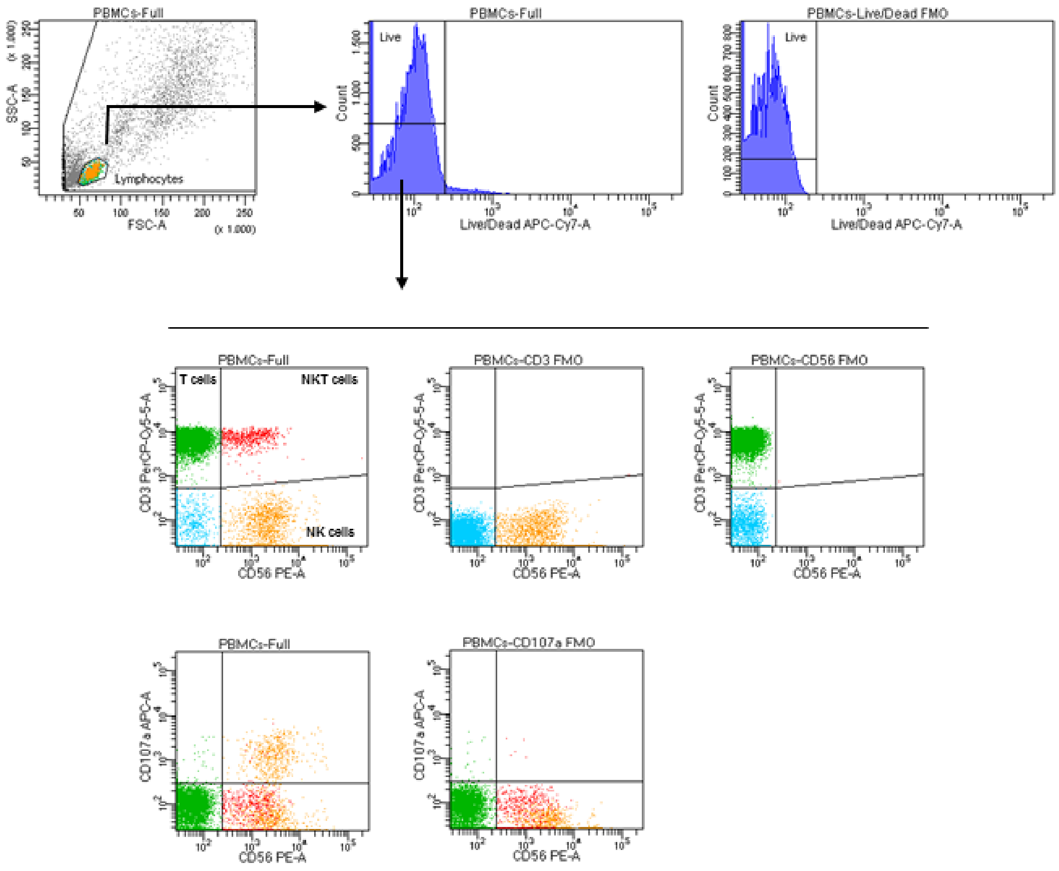

**Figure S3.** Composite ROC curves. ROC curves deriving from the percentages of NKG2D$^{pos}$ NK cells alone and from the combinations of NKG2D$^{pos}$ NK+T cells or NKG2D$^{pos}$ NK+T+NKT cells are shown. AUC= Area Under the Curve.

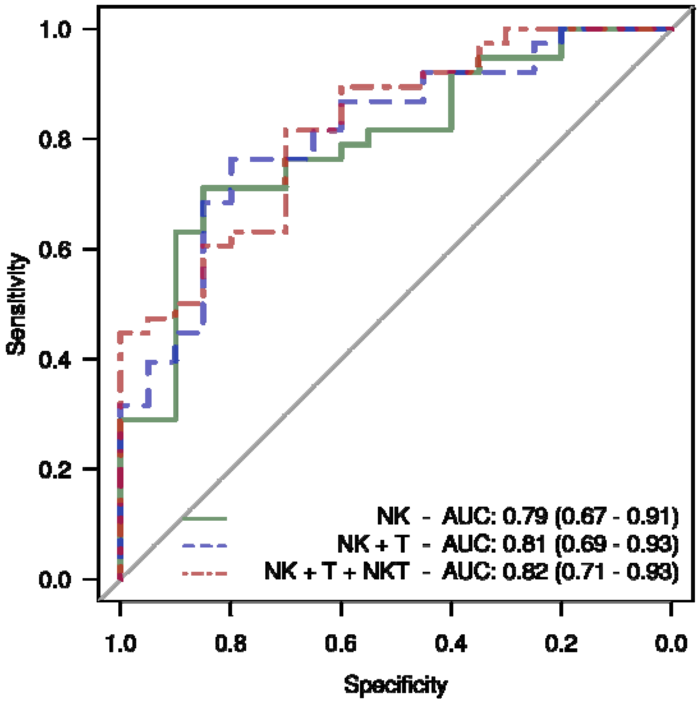

**Figure S4.** Surface markers expression in NK, NKT and T lymphocytes of BD patients according to BDCAF scores. Box plot visualization of the frequencies of CD69$^{pos}$ (**A**), NKG2A$^{pos}$ (**B**), CD16$^{pos}$ (**C**), Nkp30$^{pos}$ (**D**) and Nkp46$^{pos}$ (**E**) NK, NKT and T cells in BD patients with BDCAF = 0 *versus* BDCAF $\geq 3$ and HC. Horizontal lines show the median ± IQR. Data were analysed with Mann-Whitney U test.

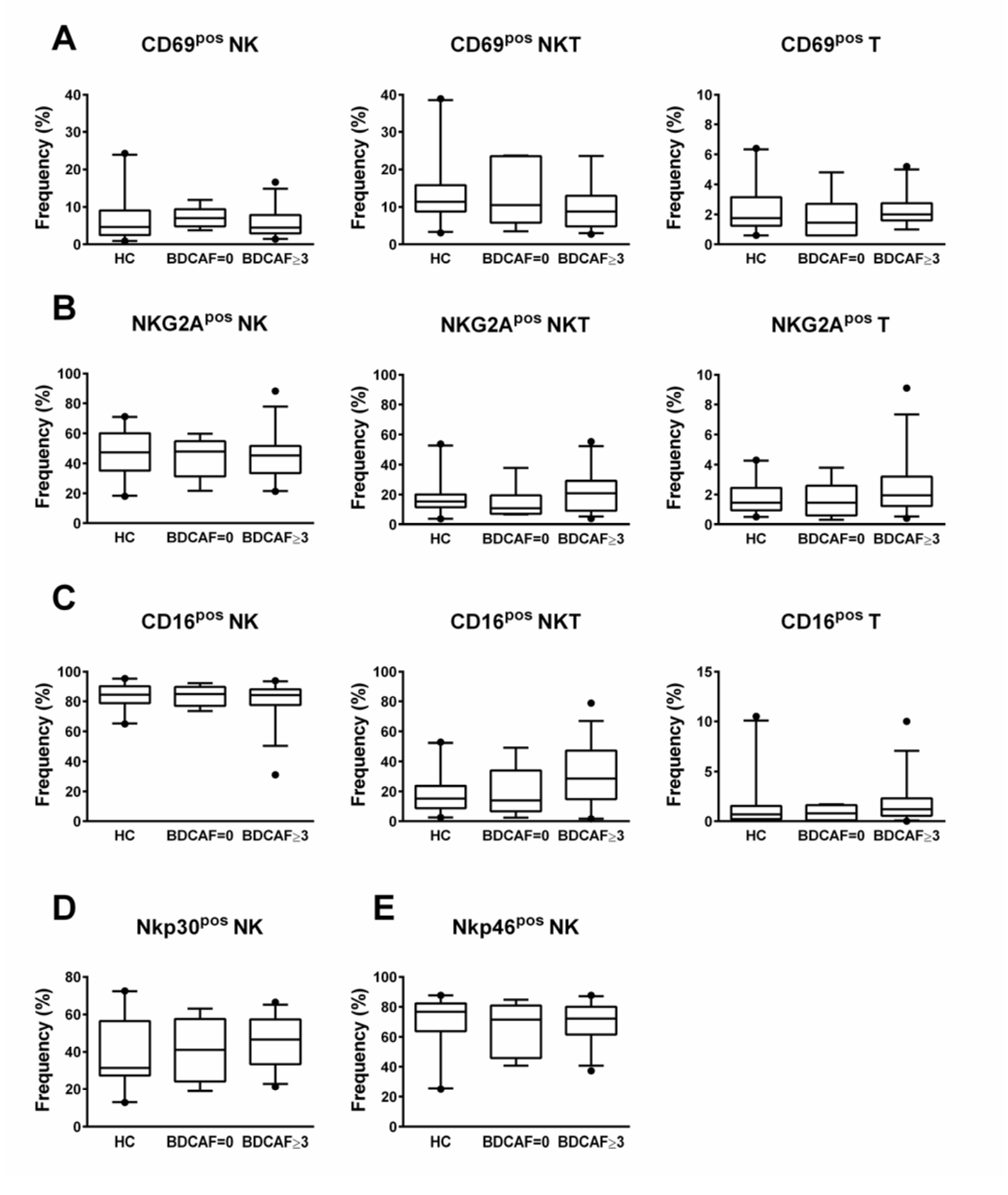

**Figure S5.** Surface markers expression in NK, NKT and T lymphocytes of BD patients according to the therapy. Dot plot visualization of the percentages of CD69$^{pos}$ (**A**), NKG2A$^{pos}$ (**B**), CD16$^{pos}$ (**C**), Nkp30$^{pos}$ (**D**) and Nkp46$^{pos}$ (**E**) cells in the NK, NKT and T lymphocyte gates determined by flow cytometry in PBMCs from BD patients classified according to presence (▲) or absence of therapy (○). Horizontal lines show the median ± IQR. Data were analysed by Mann-Whitney U test.

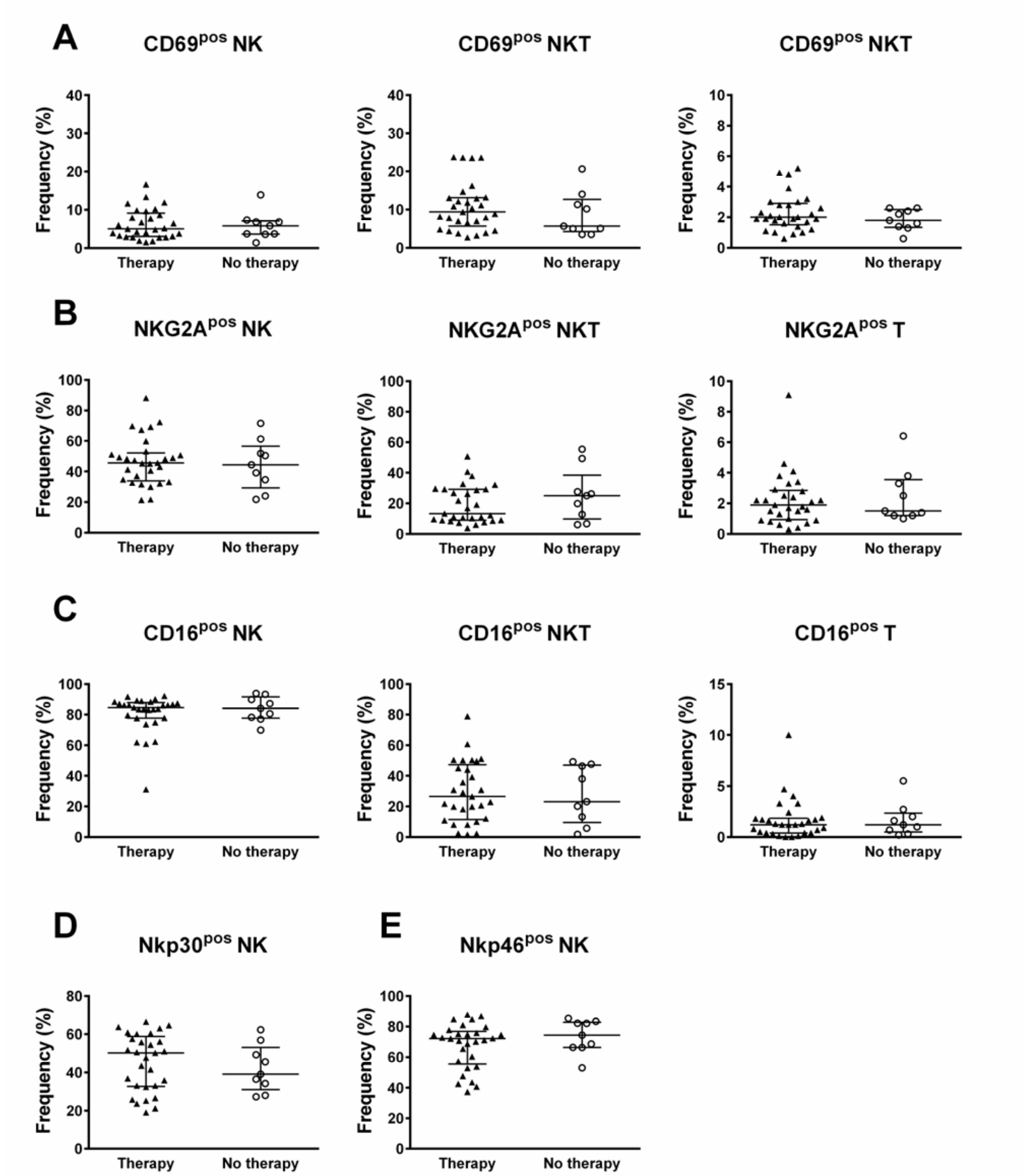

Supplement: Supplementary file 2 [file Image_1.pdf]
